# Phylogenetic Analyses of Some *Melanoleuca* Species (Agaricales, Tricholomataceae) in Northern China, With Descriptions of Two New Species and the Identification of Seven Species as a First Record

**DOI:** 10.3389/fmicb.2019.02167

**Published:** 2019-09-26

**Authors:** Jize Xu, Xiaodong Yu, Mengzhao Lu, Jiajun Hu, Odeshnee Moodley, Chunlan Zhang, Lei Gong, Yu Li

**Affiliations:** ^1^Agricultural College, Jilin Agriculture Science and Technology College, Jilin, China; ^2^School of Landscape Architecture, Changchun University, Changchun, China; ^3^Key Laboratory of Molecular Epigenetics of the Ministry of Education, Northeast Normal University, Changchun, China; ^4^Engineering Research Center of Chinese Ministry of Education for Edible and Medicinal Fungi, Jilin Agricultural University, Changchun, China; ^5^South African Department of Agriculture, Forestry and Fisheries, Pretoria, South Africa

**Keywords:** taxonomy, phylogeny, divergence time, morphological characteristics, infrageneric classifications

## Abstract

Two new species (*Melanoleuca galerina* and *M. subgrammopodia*) and seven new recorded species from northern China are described here using morphological and molecular methods. *Melanoleuca galerina* is mainly characterized by its hygrophanous pileus, decurrent lamellae, fibrous stipe and spores with round warts. Key characteristics of *M. subgrammopodia* include its discolored pileus, fibrous stipe and urticiform cystidia. The divergence time of *Melanoleuca* fungi as well as the phylogenetic relationships within this genus were analyzed using DNA sequences of the internal transcribed spacer (ITS) and the nuclear large subunit rDNA (nrLSU) gene fragments. Analyses revealed that morphological identifications and phylogenetic relationships were consistent with the results of divergence time, thereby confirming that *M. galerina* and *M. subgrammopodia* are new species.

## Introduction

Individuals from *Melanoleuca* Pat. are widely distributed throughout the world and include some edible species (Singer, 1986). Currently, Index Fungorum^1^ lists 422 validly published species within *Melanoleuca*. Whereas, Kirk et al. (2008) only accepted 50 species from this genus. In respect of China, a total of 19 *Melanoleuca* species have been reported (Bau and Li, 1999; Zhang et al., 2001; Chen, 2007; Mao, 2009; Sun et al., 2012; Wang, 2013; He et al., 2014; Yu et al., 2014; Zhao et al., 2014; Wei et al., 2015; Du et al., 2016; Tian et al., 2018). *Melanoleuca* is a genus with limited morphological characteristics. It is mainly characterized by a collybioid to tricholomatoid habit, a rarely bright-colored pileus, with warty and strongly amyloid spores and having either absent or present cystidia (Singer, 1986). The divisions among *Melanoleuca* species are vague. Different taxonomists often have their own interpretations of morphological features, resulting in different classifications of the same species based on those differing opinions (Singer, 1986; Boekhout, 1988; Hyde et al., 2013). The following division views are currently widely approved. Singer (1962, 1986) divided *Melanoleuca* into four sections primarily based on the color of the pileus or stipe and the ornamentation of spores. Boekhout (1988) focused mainly on microscopic features, on the basis of presence and shape of cystidia and divided the genus into three subgenera namely: Subg. *Melanoleuca* Boekhout, Subg. *Urocystis* Boekhout and Subg. *Macrocystis* Boekhout. Bon (1991) introduced the spore *Q* value for delimiting subsections and divided the genus into three subgenera and eight sections (Vizzini et al., 2011). Before the advent of molecular phylogenetic tools, the taxonomical units of Boekhout (1988) were most frequently used for morphological identification of *Melanoleuca*. However, Vizzini et al. (2011) divided *Melanoleuca* into two subgenera (Subg. *Urticocystis* Vizzini and Subg. *Melanoleuca* Vizzini) by constructing phylogenetic relationships with the internal transcribed spacer (ITS) sequences. Then, Yu et al. (2014) and Kalmer et al. (2018) confirmed the results of Vizzini et al. (2011). Even now, the division views proposed by Vizzini et al. (2011) are widely supported by molecular systematics (Yu et al., 2014; Kalmer et al., 2018).

Some reports have established divergence times in the fungi. For example, Hennig (1966) first recommended the use of divergence time as a universal criterion for taxa ranking. Berbee and Taylor (2010), Oberwinkler (2012), and Hibbett (2014) estimated the divergence times of Basidiomycota and Ascomycota. Time-trees indicated that Basidiomycota are estimated to have diverged around 500 million years ago (Ma), and is a sister group to Ascomycota, being of similar age. Agaricomycetes diverged about 290 Ma as estimated by Floudas et al. (2012). Zhao et al. (2016) first attempted creating a taxonomic system of fungi based on divergence time which was a reconstruction of the taxonomic system used for *Agaricus.* Furthermore, Chen et al. (2017) utilized the taxonomic system based on divergence time for reconstruction of *Agaricus* subg. *Minores*, *A.* sect. *Minores* (Fr.) Henn. Additionally, Zhao et al. (2017) proposed that the divergence time of a lineage could be used as a universal criterion for ranking taxa and estimated the divergence time of Basidiomycota. Until now, divergence time has not been introduced into studies of *Melanoleuca*, moreover, its possible usage in taxonomic studies of the genus needs further verification.

Therefore, in the present study, we made use of universal sequences in fungi (ITS + nrLSU) to estimate the divergence time of *Melanoleuca*, and discussed the infrageneric classifications of 19 species from northern China based on morphological identifications and clarified their phylogenetic relationships in order to provide a theoretical basis for the study of *Melanoleuca*.

## Materials and Methods

### Materials and Morphological Observations

All samples were collected from 2011 to 2018 from northern China, and have been deposited in the Herbarium Mycology of Jilin Agricultural University (HMJAU) and Herbarium Mycology of Jinlin Agricultural Science and Technology University (HMJU). The specific details are shown in Table 1. Pictures of the habitats were taken by a Canon 80D camera. Macroscopic features were recorded using fresh collections. Color descriptions were based upon the classifications made by Kornerup and Wanscher (1978). Dried specimens were used for microscopic observations, using 5% KOH as the floating agent, Melzer’s reagent was used to examine the presence of amyloid or dextrinoid reactions. Slices of lamellae and pileipellis were observed under the Olympus BX 53 microscope. Free hand drawings were made from all microscopic observations. Shooting and measurements of anatomical features were presented in the Cellsens Standard. The data was recorded by (a) b–c × d–e (f), n was the number of examined basidiospores and Q (length: breadth ratios) was calculated from 30 mature basidiospores of 3 basidiocarps. Cystidial shapes were described as per Vizzini et al. (2011). In addition, basidiosopres were observed under the scanning electron microscope, using the following procedure: gills were attached to specimen holders by carbon tape, coated with platinum-palladium using a Hitachi MC 1000 Ion Sputter Coater and examined with a FEI Quanta 200 FE-SEM operated at 5–10 kV.

**TABLE 1:** List of specimens used in this study.

**Species**	**Collection**	**Public database accession number**		**Geographic origin**
		**ITS**	**LSU**	
*Clitocybe subditopoda*	AFTOL-ID 533	DQ 202269	AY 691889	United States
*Leucopaxillus cerealis*	GB:0068845	KJ 417282	KJ 417198	United States
*M. angelesiana*	HMJU 00114	MK 659970	MK 660080	Heilongjiang, China
*M. angelesiana*	HMJU 00120	MK 659976	MK 660074	Heilongjiang, China
*M. arcuata*	HMJU 00118	MK 659974	MK 660076	Jilin, China
*M. arcuata*	HMJU 00141	MK 659995	MK 660054	Jilin, China
*M. arcuata*	HMJU 00149	MK 660003	MK 660046	Jilin, China
*M. arcuata*	TENN 050387	JX 429187	JX 429177	Switzerland
*M. brevipes*	HMJU 00125	MK 659980	MK 660069	Liaoning, China
*M. cinereifolia*	HMJU 00124	MK 659979	MK 660070	Jilin, China
*M. cognata*	GB 65454	JX 429190	JX 429180	Sweden
*M. communis*	HMJU 00108	MK 659964	MK 660086	Sichuan, China
*M. communis*	HMJU 00117	MK 659973	MK 660077	Jilin, China
*M. communis*	HMJU 00139	MK 659993	MK 660056	Liaoning, China
*M. communis*	HMJU 00143	MK 659997	MK 660052	Jilin, China
*M. communis*	HMJU 00144	MK 659998	MK 660051	Liaoning, China
*M. communis*	HMJU 00146	MK 660000	MK 660049	Jilin, China
*M. dryophila*	HMJU 00121	MK 659977	MK 660073	Jilin, China
*M. dryophila*	HMJU 00123	MK 659978	MK 660071	Jilin, China
*M. dryophila*	HMJU 00126	MK 659981	MK 660068	Liaoning, China
*M. dryophila*	HMJU 00140	MK 659994	MK 660055	Liaoning, China
*M. dryophila*	HMJU 00145	MK 659999	MK 660050	Liaoning, China
*M. exscissa*	HMJU 00107	MK 659963	MK 660087	Liaoning, China
*M. exscissa*	TENN 057720	JX 429191	JX 429184	Germany
*M. exscissa*	GB 65455	JX 429192	JX 429178	Sweden
*M. friesii*	HMJU 00129	MK 659983	MK 660065	Liaoning, China
*M. friesii*	HMJU 00136	MK 659990	MK 660058	Qinghai, China
*M. friesii*	HMJU 00137	MK 659991	MK 660057	Gansu, China
*M. friesii*	HMJU 00142	MK 659996	MK 660053	Liaoning, China
*M. galerina*	HMJU 00103	MK 583563	MK 660072	Jilin, China
*M. galerina*	HMJAU 48287	MN173526	MN173536	Jilin, China
*M. grammopodia*	TENN 037162	JX 429194	JX 429179	Poland
*M. griseobrunnea*	HMJU 00134	MK 659988	MK 660060	Gansu, China
*M. herrerae*	JCB 3445	JX 429224	JX 429164	Mexico
*M. herrerae*	TXLM AME 1282	JX 429199	JX 429165	Mexico
*M. jaliscoensis*	GL 50	JX 429222	JX 429176	Mexico
*M. jaliscoensis*	MRSJ 966	JX 429218	JX 429173	Mexico
*M. leucopoda*	HMJU 00109	MK 659965	MK 660085	Neimenggu, China
*M. leucopoda*	HMJU 00115	MK 659971	MK 660079	Heilongjiang, China
*M. leucopoda*	HMJU 00130	MK 659984	MK 660064	Jilin, China
*M. leucopoda*	HMJU 00147	MK 660001	MK 660048	Liaoning, China
*M. longisterigma*	ENCB Guzman 11494	JX 429212	JX 429171	Mexico
*M. melaleuca*	CBS 230.46	MH 856170	MH 867694	France
*M. microcephala*	HMJU 00132	MK 659986	MK 660062	Gansu, China
*M. microcephala*	HMJU 00133	MK 659987	MK 660061	Gansu, China
*M. microcephala*	HMJU 00138	MK 659992	MK 660045	Gansu, China
*M. nivea*	HMJU 00148	MK 660002	MK 660047	Jilin, China
*M. paedida*	HMJU 00135	MK 659989	MK 660059	Gansu, China
*M. polioleuca*	GB 65471	JX 429196	JX 429181	Sweden
*M. porphyropoda*	HMJU 00110	MK 659966	MK 660084	Neimenggu, China
*M. porphyropoda*	HMJU 00116	MK 659972	MK 660078	Jilin, China
*M. pseudopaedida*	HMJU 00155	MK 978844	MK 979277	Qinghai, China
*M. strictipes*	GB 65498	JX 429116	JX 429162	Sweden
*M. stridula*	HMJU 00105	MK 660004	MK 660089	Heilongjiang, China
*M. stridula*	HMJU 00111	MK 659967	MK 660083	Neimenggu, China
*M. stridula*	HMJU 00127	MK 659982	MK 660067	Jilin, China
*M. subgrammopodia*	HMJAU 48288	MN173526	MN173526	Jilin, China
*M. subgrammopodia*	HMJU 00104	MK 583562	MK 660066	Jilin, China
*M. verrucipes*	HMJU 00131	MK 659985	MK 660063	Liaoning, China
*Melanoleuca sp.*	HMJU 00119	MK 659975	MK 660075	Jilin, China
*Mycena plumbea*	AFTOL-ID 1631	DQ 494677	DQ 470813	United States
*Pluteus romellii*	AFTOL-ID 625	AY 854065	AY 634279	United States
*Suillus pictus*	AFTOL-ID 717	AY 854069	AY 684154	United States

### DNA Extraction, PCR, Purification and Sequencing

Genomic DNA was extracted from the dried specimens following the procedure described by Zhao et al. (2011). Polymerase chain reaction amplified sequences of the ITS and the nrLSU regions. Primers ITS1 and ITS4 (White et al., 1990) were used for the ITS region while primers LROR (Rehner and Samuels, 1994) and LR7 (Vilgalys and Hester, 1990) were used for the nrLSU region. The total volume of the PCR amplification reaction system was 50 μL containing of 10 μL of 5 × PCR buffer (Dingguo, Beijing, China), 4 μL of 200 μmol/L deoxynucleoside triphosphates, 1 μL of 200 μmol/L each primer, 5 U of Taq DNA polymerase and 10 μL of template DNA. The program parameters were set as follows, for ITS: initial denaturation at 94°C for 4 min; repeated for 30 cycles, denaturation at 94°C for 1 min, with annealing at 55°C for 1 min, extension at 72°C for 1 min, left at 72°C for 5 min and saved at 4°C; nrLSU: initial denaturation at 94°C for 4 min, repeated for 30 cycles, denaturation at 94°C for 90 s, with annealing at 55°C for 90 s, extension at 72°C for 90 s, left at 72°C for 5 min and saved at 4°C.

The products of PCR amplification were purified with the *EasyPure* Plasmid MiniPrep Kit (TransGen Biotech Co., Ltd., Beijing, China.), and resolved on a 1.0% agarose gel and subsequently submitted for sequencing (sequencing was completed by BGI Co., Ltd., Beijing, China).

### Phylogenetic Analysis

A total of 110 sequences (ITS and nrLSU) representing 29 species were incorporated in the phylogenetic analyses, of which 26 sequences were retrieved from GenBank. *Clitocybe subditopoda* was used as the outgroup. Detailed specimen information appears on Table 1. All the sequences were aligned in Clustal X 2.1 (Lin et al., 2017). The conservative region was selected in Gblock^2^ and the vacancy gap in the data were treated as missing data (Talavera and Castresana, 2007). Saturation was tested using DAMBE 5.2 (Posada and Crandall, 1998) (model = test by Xia, 2013). MrModel Test 2.3 were used to select the fragment models (Wilgenbusch and Swofford, 2003; Nylander, 2004). The best model was used (ITS-nrLSU: GC) to construct a Maximum likelihood (ML) tree with PhyML (Guindon et al., 2009). The ML tree was evaluated by bootstrap analysis with 1000 replicates (Stamatakis, 2006). Bootstrap values greater than or equal to 60% were indicated along nodes.

### Divergence-Time Analysis

Sequences incorporated in phylogenetic analyses were used to estimate the divergence time. All the sequences were aligned by MEGA v.7.0^3^. Four species of Tricholomataceae were used as the outgroup. We used divergence times of *Boletales* (189 ± 20Ma), *Archaeomarasmius leggetti* (90 Ma), *Quatsinoporises cranhanaii* (125 Ma) and *Mycena plumbea* (90 ± 30 Ma) as calibration points (Hibbett et al., 1997; Smith et al., 2004; Feng, 2012). Divergence time was estimated in BEAST v.2.5.1. The best substitution model for each partition was inferred with the program MrModeltest 2.2 (Nylander, 2004): GTR + G + I for ITS and nrLSU. The number of substitution rate categories and Gamma shape parameters were 4.0362 and 4.0343, respectively. BEAUti v.2.5.1^4^ was used to construct an XML file. The Relaxed clock model was selected according to the ESS value exceeding 200. Substitution models were independently estimated for each gene partition. The Yule speciation prior set was used to estimate the divergence time and the corresponding credibility intervals were constructed using treeModel. We ran an independent Monte Carlo Markov Chains (MCMC) of 10 million generations, logging states every 10,000 generations.

The checking for convergence and mixing of Log files were completed in Tracer v1.6^5^. Tree files were summarized by the TreeAnnotator v.2.5.1., discarding 10% of states as burn-in and annotating clades with ≥0.8 posterior probability, and the maximum-clade-credibility tree (MCC) was generated. The resulting files were viewed using Figtree v.1.4^6^.

## Results

### Taxanomy

**FIGURE 1:**
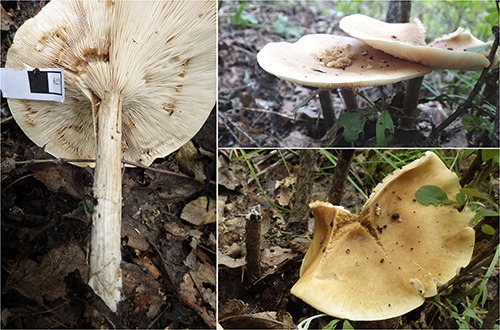
Basidiomata of *Melanoleuca galerina* (Holotype, HMJAU48281).

**FIGURE 2:**
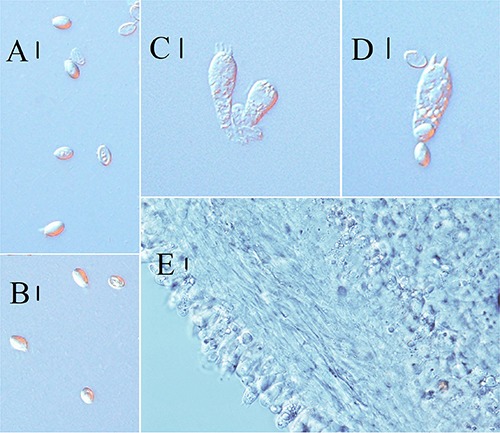
Microscopic characteristics of *Melanoleuca galerina* (Holotype, HMJAU48281). **(A,B)** Basidiospores; **(C,D)** Basidia; and **(E)** Trama (**A,B** = 5 μm; **C–E** = 10 μm).

**FIGURE 3:**
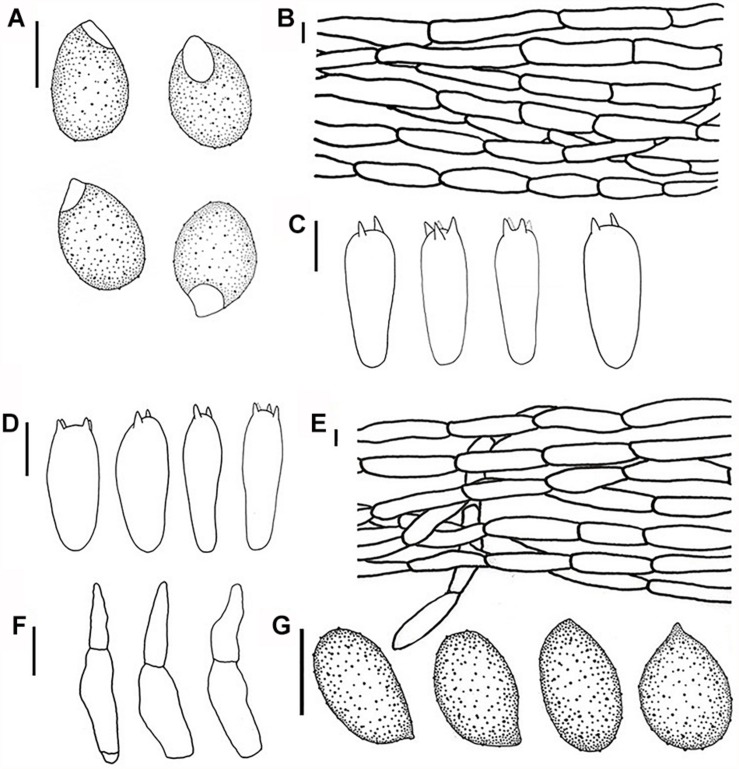
Line drawings: **(A–C)** *Melanoleuca galerina* (Holotype, HMJAU48281), **(A)** Basidiospores; **(B)** Pileipellis; **(C)** Basidia; **(D–G)** *Melanoleuca subgrammopodia* (Holotype, HMJAU48287), **(D)** Basidia; **(E)** Pileipellis; **(F)** Cheilocystidia; and **(G)** Basidiospores (**A,G** = 5 μm; **B–E**, *F* = 10 μm).

**FIGURE 4:**
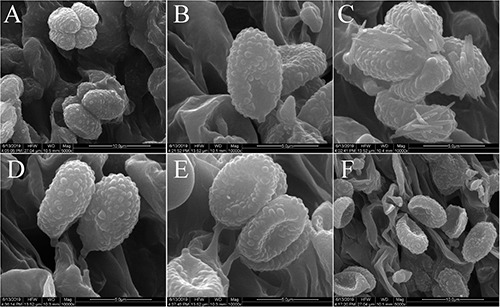
Scanning electron microscope images of basidiospores: **(A–C)** *Melanoleuca galerina* (Holotype, HMJAU48281); **(D–F)** *M. subgrammopodia* (Holotype, HMJAU48287).

***Melanoleuca galerina*** YL and JX, sp. nov. (Figures 1, 2, 3A–C, 4A–C)

MycoBank no.: MB 830397

Diagnosis: Pileus 8.5–9.8 cm, plane or slightly depressed at disc, pale when young, pale brown (5C4) to margin at the later stage, margin pale (1A2), surface hygrophanous, dehiscent in the center in the mature period. Lamellae white (1A2) when young, pale rose pink (4A2) in the mature period. Stipe 10–13 × 1.3–1.7 cm, longitudinally fibrous striate, slightly distorted, hollow. Cystidia absent. Clamp connections absent. Basidiospores (6.7) 7.1–8.4 × (3.5) 4.4–5.4 μm, amyloid.

Type: China: Jilin Province, Jilin City, Zuojia Town, 44°05′N, 126°05′E, 7 September 2016, Jize Xu, HMJAU48281 (Holotype).

Etymology: referring to the basidiospores, which are similar to *Galerina*.

Pileus 8.5–9.8 cm, plane or slightly depressed at disc, near round, light brown (5C4) in the center, slightly paling toward the margin, surface hygrophanous, smooth, not viscid when moist, margin pale (1A2), even, slightly cracked when matured. Lamellae 0.3–0.4 cm broad, decurrent, white (1A2) at first, pale rose pink (4A2) in the mature period, 19 lamellae/cm in the edge of the pileus. Stipe 10–13 × 1.3–1.7 cm, white (2A2) to creamy (29A2), pale brown (5C4) when moist, subcylindrical, longitudinally fibrous striate, slightly distorted, subequal, slightly attenuated in the middle, with poor pallid (1A1) tomentum toward the base, hollow when matured. Context 0.5 cm thick at pileus center, dirty white (28A2) to pale brown (5C4), with indistinct or slight fungoid smell and mild taste. Spore print white.

Basidiospores (6.7) 7.1–8.4 × (3.5) 4.4–5.4 μm, *Q* = (1.43) 1.51–1.89 (2.16), (*n* = 30), oblong-ellipsoid, hyaline, some with one oil drop, few with encrusted crystals on the surface, ornamented with round warts, warts up to 0.5 μm wide and 0.3–0.6 μm high, amyloid. Basidia 31–36 (39) × (7.4) 7.8–8.5 μm, clavate to subcylindrical, slightly broadened at apex, with two or four sterigmata, sterigmata up to 2.5–4.0 μm long. Cheilocystidia and pleurocystidia absent. Trama regular, hyphae 9.5–12 μm wide, cylindrical, thin-walled, hyaline. Pileipellis a cutis of radially parallel, thin-walled, dense hyphae, hyphae 10–14 μm wide, cylindrical, not or slightly constricted at the septa, hyaline. Clamp connections absent.

Habitat and distribution: Scattered on grass. Known from Jilin Province in China.

Additional specimens examined: China: Jilin Province: Jilin City, Zuojia Town, 44°05′N, 126°05′E, 7 September 2016, Jize Xu, HMJU00103.

Notes: *Melanoleuca galerina* is mainly characterized as having medium to large basidiomata, with a hygrophanous pileus, decurrent lamellae and a fibrous stipe which is hollow at maturity. It belongs to the subgenus *Melanoleuca* which has a key characteristic in that it lacks cystidia (Boekhout, 1988). In the subgenus *Melanoleuca*, *M. galerina* differs from *M. ustaliformis* Murrill, *M. melaleuciformis* Murrill, *M. subfulvidisca* Murrill and *M. westiana* Murrill in having a pale brown pileus and decurrent lamellae (Hesler, 2013b). *Melanoleuca compressipes* Murrill, *M. albissima* Murrill and *M. watsonii* Murrill are easily distinguished from *M. galerina* by their glabrous stipes (Murrill, 1940; Hesler, 2013b), apart from this, the spores in *M. watsonii* are much smaller than those of *M. galerina* (4–5 × 2.3–2.5 μm in *M. watsonii*, 7.2–8.3 × 4.3–4.9 μm in *M. galerina*). *Melanoleuca compressipes* differs from *M. galerina* in its sordid-white to dark-brown lamellae. In gross morphology, *M. galerina* is exceedingly similar to *M. clelandii* Grgur., but *M. clelandii* differs from *M. galerina* in having a larger basidiomata, with a pileus diameter up to 15.2 cm while the pileus diameter of *M. galerina* is approximately 9 cm (Grgurinovic, 1985).

**FIGURE 5:**
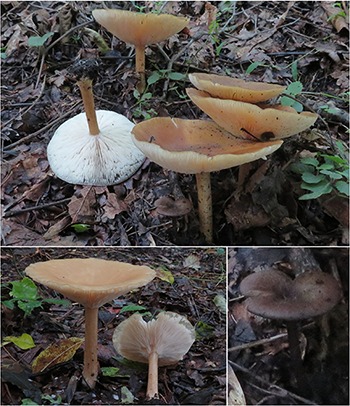
Basidiomata of *Melanoleuca subgrammopodia* (Holotype, HMJAU48287).

**FIGURE 6:**
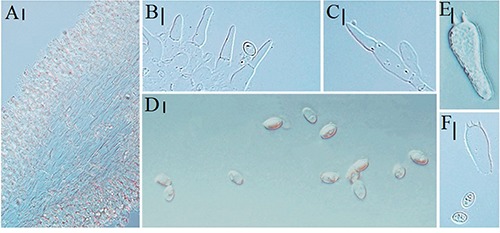
Microscopic characteristics of *Melanoleuca subgrammopodia* (Holotype, HMJAU48287). **(A)** Trama; **(B,C)** Cheilocystidia; **(D)** Basidiospores; and **(E,F)** Basidia (**A–C** = 10 μm; **D** = 5 μm; **E,F** = 10 μm).

***Melanoleuca subgrammopodia*** YL and JX, sp. nov. (Figures 3D–G, 4D–F, 5, 6).

MycoBank no.: MB830552

Diagnosis: Pileus 10–13 cm, depressed in the center, dark brown (9F6) at first, then becoming lighter, margin incurved when young, applanated after maturity. Lamellae 0.2–0.3 cm broad, white (1A2), separated from the edge of pileus after maturity. Stipe 6.5–7.5 × 0.7–0.9 cm near black (7F3) when young, light brown (6C6) when mature, longitudinally fibrous striate. Clamp connections absent. Cheilocystidia urticiform, of *brevipes*-type, septate, without crystals at the apex. Basidiospores (6.2) 7.2–8.3 (8.6) × 4.3–4.9 (5.3) μm, amyloid.

Type: China: Jilin Province, Jiaohe City, Hongyegu, 43°40′N, 127°04′E, 6 September 2018, Jize Xu, Jiajun Hu, HMJAU48287 (Holotype).

Etymology: refers to the longitudinally fibrous striate stipe.

Pileus 10–13 cm, depressed in the center, shallow funnel, dark brown (9F6) at first, then becoming lighter, pale brown (5B3) in the mature period, surface dry, smooth, not viscid when moist, margin even, slightly cracked, incurved when young, applanated after maturity. Lamellae 0.2–0.3 cm broad, adnate to decurrent, white (1A2), 9–11 lamellae/cm in the edge of the pileus, with intercalated lamellulae, separated from the edge of pileus after maturity. Stipe 6.5–7.5 × 0.7–0.9 cm, near black (7F3) when young, light brown (6C6) in the mature period, broadened at base, longitudinally fibrous striate. Context 0.2 cm thick at pileus center, dirty white (6B1), smell and taste indistinct. Spore print white.

Basidiospores (6.2) 7.2–8.3 (8.6) × 4.3–4.9 (5.3) μm, *Q* = (1.26) 1.46–1.76 (1.81), (*n* = 30), elliptical to subovoid, most with one oil drop, hyaline, ornamented with warts, warts round, up to 0.3 μm wide and 0.2–0.6 μm high, amyloid. Basidia 39–43 × 9.2–10.3 μm, clavate, slightly broadened at apex, with two or four sterigmata, sterigmata up to 1.5–4 μm long. Cheilocystidia urticiform, 38–50 × 6–10 μm, of *brevipes*-type, attenuated toward the apex, septate, without crystals at apex, hyaline. Pleurocystidia rare, similar to cheilocystidia. Trama regular, hyphae 11–13 μm cylindrical, thin-walled, hyaline. Pileipellis a cutis of radially parallel, thin-walled, dense hyphae, hyphae 11–14 μm wide, cylindrical, not or slightly constricted at the septa, hyaline. Clamp connections absent.

Habitat and distribution: Scattered on grass. Known from Jilin Province in China.

Additional specimens examined: China: Jilin Province, Jiaohe City, Hongyegu, 43°40′N, 127°04′E, 6 September 2018, Jize Xu, Jiajun Hu, HMJU00104.

Notes: The main characteristics of *M. subgrammopodia* are its larger basidiocarps, white lamellae, short stipe and urticiform cystidia. *Melanoleuca subgrammopodia* is related to members of the section *Grammpodiae* in the subgenus *Urticocystis* (Boekhout, 1988). *Melanoleuca floridana* Murrill is distinct from *M. subgrammopodia* in having a subtomentose pileus and stipe which are basal part clavate (Hesler, 2013a). *Melanoleuca juliannae* Rimóczi, Antonín, L. Nagy and Tomšovský is characterized by a violaceous-blue stipe and two types of cheilocystidia (the *exscissa*-type is most common while the *brevipes*-type is less frequent) (Vladimír et al., 2014). However, there is only one type of cheilocystidia in *M. subgrammopodia* (*brevipes*-type). *Melanoleuca subgrammopodia* is also close to *M. subacris* Murrill and *M. subcylindrispora* Murrill, *M. subacris* is distinct from *M. subgrammopodia* in having a uniform white pileus. The main characteristic of *M. subcylindrispora* are its subcylindrical spores, but the spores of *M. subgrammopodia* are elliptical to subovoid (Hesler, 2013b).

### New Recorded Species in China

*Melanoleuca griseobrunnea* Antonín, Ďuriška and Tomšovský, *M. pseudopaedida* Bon, *M. angelesiana* A. H. Smith, *M. microcephala* (P. Karst.) Singer, *M. communis* Sánchez-García and J. Cifuentes, *M. cinereifolia* (Bon) Bon, *M. nivea* Métrod ex Boekhout are first recorded in China.

**FIGURE 7:**
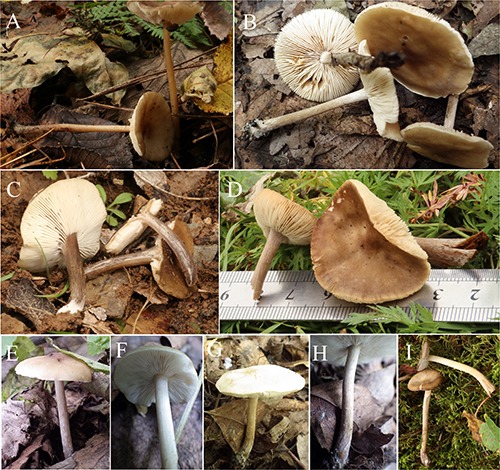
Basidiomata: **(A)** *Melanoleuca angelesiana* (HMJU00120); **(B)** *M. cinereifolia* (HMJU00124); **(C)** *M. griseobrunnea* (HMJU00134); **(D)** *M. pseudopaedida* (HMJU00155); **(E,F)** *M. nivea* (HMJU00148); **(G,H)** *M. communis* (HMJU00139); and **(I)** *M. microcephala* (HMJU00132).

**FIGURE 8:**
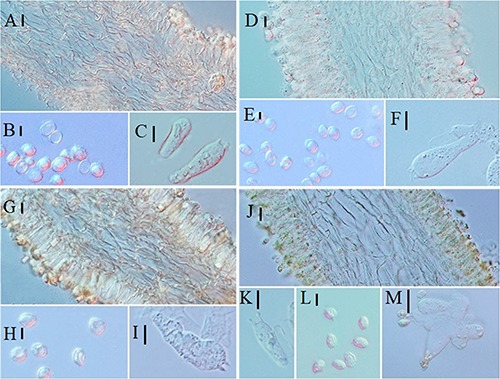
Microscopic characteristics: **(A–C)** *Melanoleuca pseudopaedida* (HMJU00155), **(A)** Trama; **(B)** Basidiospores; **(C)** Basidia; **(D–F)** *M. microcephala* (HMJU00132), **(D)** Trama; **(E)** Basidiospores; **(F)** Basidia; **(G–I)** *M. angelesiana* (HMJU00120), **(G)** Trama; **(H)** Basidiospores; **(I)** Basidia; **(J–M)** *M. cinereifolia* (HMJU00124), **(J)** Trama; **(K)** Basidia; **(L)** Basidiospores; **(M)** Cheilocystidia (**A,C,D,F,G,I,M** = 10 μm; **B,E,H,J,K,L** = 5 μm).

**FIGURE 9:**
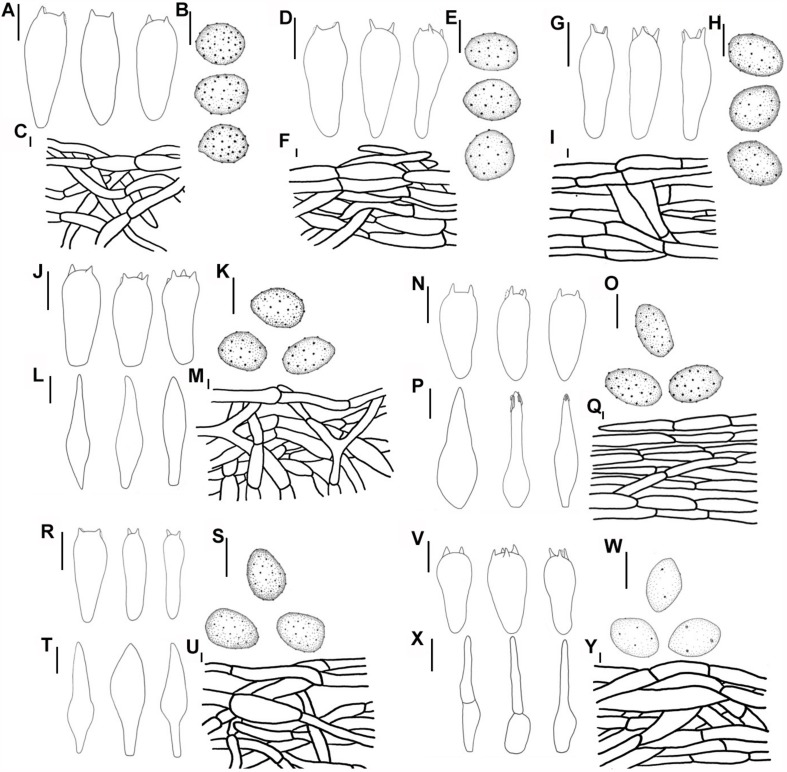
Line drawings: **(A–C)** *M. pseudopaedida* (HMJU00155), **(A)** Basidia; **(B)** Basidiospores; **(C)** Pileipellis; **(D–F)** *M. microcephala* (HMJU00132), **(D)** Basidia; **(E)** Basidiospores; **(F)** Pileipellis; **(G–I)** *M. angelesiana* (HMJU00120), **(G)** Basidia; **(H)** Basidiospores; **(I)** Pileipellis; **(J–M)** *M. communis* (HMJU00139), **(J)** Basidia; **(K)** Basidiospores; **(L)** Cheilocystidia; **(M)** Pileipellis; **(N–Q)** *M. nivea* (HMJU00148), **(N)** Basidia; **(O)** Basidiospores; **(P)** Cheilocystidia; **(Q)** Pileipellis; **(R–U)** *M. cinereifolia* (HMJU00124), **(R)** Basidia; **(S)** Basidiospores; **(T)** Cheilocystidia; **(U)** Pileipellis; **(V–Y)** *M. griseobrunnea* (HMJU00134), **(V)** Basidia; **(W)** Basidiospores; **(X)** Cheilocystidia; **(Y)** Pileipellis (**A,D,G,J,L,N,P,R,T,V,X** = 10 μm; **B,C,E,F,H,I,K,M,O,Q,S,U,W,Y** = 5 μm).

**FIGURE 10:**
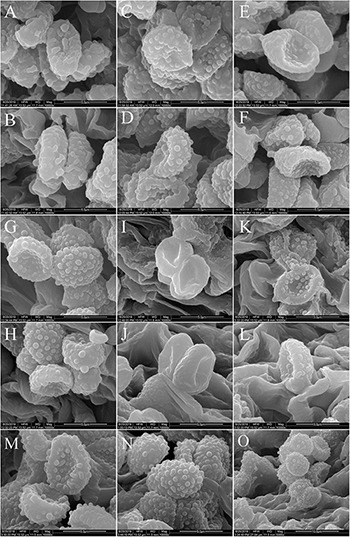
Scanning electron microscope images of basidiospores: **(A,B)** *M. cinereifolia* (HMJU00124); **(C,D)** *M. angelesiana* (HMJU00120); **(E,F)** *M. communis* (HMJU00139); **(G,H)** *M. microcephala* (HMJU00132); **(I,J)** *M. griseobrunnea* (HMJU00134); **(K,L)** *M. nivea* (HMJU00148); **(M–O)** *M. pseudopaedida* (HMJU00155).

***Melanoleuca angelesiana*** A. H. Smith, Mycologia 36: 252. 1944 (Figures 7A, 8G–I, 9G–I, 10C,D).

Pileus 5–7 cm diameter, depressed at disc, dull olive brown, surface glabrous, dry, smooth, subviscid when moist, margin incurved, becoming plane or uplifted, paler grayish brown. Lamellae adnate, pale gray to pallid or white, moderately broad, narrowed toward the margin, 8–9 lamellae/cm in the edge of the pileus, with intercalated lamellulae, the edges even and staining brownish where bruised. Stipe 5–6 × 1–1.2 cm, surface concolorous with the pileus or paler, slightly flared at the base, longitudinally fibrous striate, glabrous, hollow when matured. Context 2–3 mm thick at pileus center, watery gray when moist, pallid when faded, odor none, taste mild. Spore print white.

Basidiospores 6.5–7.7 (7.9) × 4.5–5.5 (6.1) μm, *Q* = (1.27) 1.34–1.70 (1.79), (*n* = 30), elliptical, covered with strongly amyloid minute warts. Basidia 34–42 × 9.1–12 μm, clavate, with four sterigmata. Pleurocystidia and cheilocystidia absent. Trama interwoven, inamyloid. Pileipellis a cutis composed of interwoven to radial, thin-walled, homogeneous, dense hyphae, hyphae 7–13 μm wide, cylindrical, not or slightly constricted at the septa, hyaline.

Specimens examined: China: Heilongjiang Province, Yichun City, 12 September 2017, Jize Xu, HMJU00120; same location, 11 September 2017, Jize Xu, HMJU00114.

Habitat and distribution: Solitary, on ground of coniferous mixed forest. Known from China and America.

Notes: *M. angelesiana* is placed in clade A and belonging to subgenus *Urticocystis*. The Chinese collection shares very similar morphological features and DNA sequence (ITS and nrLSU). Differing from holotype (Smith, 1944), the material from Heilongjiang produces a dry pileus which is not umbonate.

**FIGURE 11:**
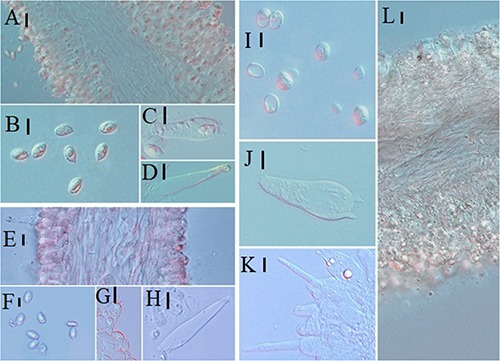
Microscopic characteristics: **(A–D)** *Melanoleuca communis* (HMJU00139), **(A)** Trama; **(B)** Basidiospores; **(C)** Basidia; **(D)** Cheilocystidia; **(E–H)** *M. nivea* (HMJU00148), **(E)** Trama; **(F)** Basidiospores; **(G)** Basidia; **(H)** Cheilocystidia; **(I–L)** *M. griseobrunnea* (HMJU00134), **(I)** Basidiospores; **(J)** Basidia; **(K)** Cheilocystidia; **(L)** Trama (**A,C,D,E,G,H,J,K,L** = 10 μm; **B,F,I** = 5 μm).

***Melanoleuca communis*** Sánchez-García & J. Cifuentes, Revista Mexicana de Micologia Suplemento-Micologia 116. 2013 (Figures 7G,H, 9J–M, 10E,F, 11A–D).

Pileus 2.8–16 cm diameter, plane to plane-convex, sometimes umbonate, brown slightly paler or yellowish-brown toward the margin, surface moist, not viscid, smooth, margin slightly incurved. Lamellae sinuate or adnate, white to yellowish, 6–8 lamellae/cm in the edge of the pileus, with intercalated lamellulae, edges jagged. Stipe 4–17 × 0.4–1.2 (1.8) cm, white to pale yellow, cylindrical to slightly attenuated toward the base, with yellowish longitudinal stripes, fleshy-fibrous, slightly whitish pruinose, solid. Context 1–2 mm thick at pileus center, white, sometimes brown-beige or grayish, smell sweetish, pleasant, sometimes absent, taste farinaceous, bitter, sometimes absent. Spore print white.

Basidiospores (6.5) 6.7–8.9 (9.4) × 4.1 (4.9)–5.6 μm, *Q* = 1.41–1.64 (1.68), (*n* = 30), elliptical to oblong, ornamented with amyloid, isolated warts, hyaline. Basidia (18) 21–31 (39) × (5) 8–10 μm, clavate, with four sterigmata. Cheilocystidia 51–67 × 11–16 μm, fusiform, sometimes lageniform usually with crystals at the apices. Pleurocystidia similar to cheilocystidia. Trama regular, inamyloid. Pileipellis a cutis constituted of interwoven, thin-walled, dense hyphae, hyphae 6–18 μm wide, cylindrical, not or slightly constricted at the septa, hyaline.

Specimens examined: China: Jilin Province, Jilin City, Zuojia Town, 5 September 2016, Jize Xu, HMJU00117; same location, 27 July 2016, Jize Xu, HMJU00146; same location, 27 July 2017, Jize Xu, HMJU00143; Liaoning Province, Fuxin City, Fumeng Town, Haitang Mountain, 6 August 2016, Jize Xu, HMJU00139, HMJU00144.

Habitat and distribution: Scattered on grass. Known from China and Mexico.

Notes: *M. communis* is included in clade B and belonging to subgenus *Melanoleuca*. Differing from holotype (Sánchez-García et al., 2013), the edges of lamellae for Chinese collections are jagged, and the stipes are slightly whitish pruinose.

***Melanoleuca cinereifolia*** (Bon) Bon, Documents Mycologiques 9: 71. 1978 (Figures 7B, 8J–M, 9R–U, 10A,B).

Pileus 3.5–5.0 cm diameter, plane-convex to plane, cafe-gray, surface smooth, moist not viscid, margin straight. Lamellae sinuate, adnate, white, 12–13 lamellae/cm in the edge of the pileus, with intercalated lamellulae. Stipe 4 × 0.3 cm, surface concolorous with the pileus, cylindrical, longitudinally striate, fleshy-fibrous, pruinose or finely floccose at base, solid. Context 1.5–2.5 mm thick at pileus center, cafe-gray to chocolate color, odor none, taste absent. Spore print white.

Basidiospores 7.2–8.4 × 4.3–5.2 μm, *Q* = 1.42–2.00, (*n* = 30), elliptical to oblong, ornamented with warts, hyaline, amyloid; Basidia (20) 23–31 (33) × 8 (10) μm, clavate, with four sterigmata; Cheilocystidia (41) 49–59 (64) × (8) 10–12 (13) μm, fusiform to lageniform, with crystals at the apices. Pleurocystidia similar to cheilocystidia. Trama regular, inamyloid. Pileipellis tomentum, a cutis composed of thin-walled, dense hyphae, hyphae 6–18 μm wide, cylindrical, not or slightly constricted at the septa, hyaline.

Specimens examined: China: Jilin Province, Jilin City, Zuojia Town, 7 September 2016, Jize Xu, HMJU00124.

Habitat and distribution: Solitary, on grass of forest. Known from China and Mexico.

Notes: *M. cinereifolia* is in clade B and belonging to subgenus *Melanoleuca*. Differing from interpretations of Sánchez-García et al. (2013), the material from Jilin produces a solid stipe which is pruinose or finely floccose at base.

***Melanoleuca griseobrunnea*** Antonín, Ďuriška & Tomšovský, Plant Systematics and Evolution 303: 1195. 2017 (Figures 7C, 9V–Y, 10I,J, 11I–L).

Pileus 2.0–3.5 cm diameter, applanate with or without a shallow central depression and with small central umbo, dark gray-brown, sometimes paler toward margin, surface smooth, glabrous to finely tomentose, not viscid when moist, slightly pruinose at margin, margin slightly incurved. Lamellae adnexed to slightly decurrent with tooth, pale dirty cream, 16–19 lamellae/cm in the edge of the pileus, with intercalated lamellulae. Stipe 2.5–4.5 × 0.3–0.5 cm, gray brown, apex slightly paler, cylindrical, slightly clavate at base, pruinose or finely floccose at apex. Context 0.7–0.9 mm thick at pileus center, whitish to grayish brownish, smell fungoid, taste mild with unpleasant aftertaste. Spore print whitish.

Basidiospores (5.7) 6.3–7.6 × (3.9) 4.1–5.4 μm, *Q* = 1.40–1.51, (*n* = 30), elliptical, ovoid, with verruculose ornamentation, amyloid. Basidia 18–42 × 6.0–12 μm, clavate, subcylindrical or subfusoid, with four sterigmata. Cheilocystidia 25–46 × 6.0–10.5 μm, urticiform of both the exscissa- and brevipes-type. Pleurocystidia similar to cheilocystidia. Trama regular, inamyloid. Pileipellis a cutis constituted of subradially arranged, thin-walled hyphae, hyphae 5–13 μm wide, cylindrical, hyaline.

Specimens examined: China: Ningxia Hui Autonomous Region, Yinchuan City, Suyukou National Forest Park, 13 August 2018, Jize Xu, HMJU00134.

Habitat and distribution: Scattered on sandy soil. Known from China and Korea.

Notes: *M. griseobrunnea* is placed in clade A and belonging to subgenus *Urticocystis*. The species is related to *M. porphyropoda*, but *M. griseobrunnea* is distinguished by having urticiform cystidia (Yu et al., 2014). Differing from holotype (Antonín et al., 2017), the edges of lamellae for Chinese collections are jagged, and the stipes are slightly whitish pruinose.

***Melanoleuca microcephala*** (P. Karst.) Singer, Cavanillesia 7: 123. 1935 (Figures 7I, 8D–F, 9D–F, 10G,H).

Pileus 1.8–2.5 cm diameter, plane or slightly depressed at disc, round or nearly circular, pale brown, sometimes lighter toward margin, surface dry, smooth, margin pale, incurved, slightly cracked. Lamellae adnate to slightly decurrent, white, 20–25 lamellae/cm in the edge of the pileus, with intercalated lamellulae. Stipe 5.3–6.3 × 0.3–0.5 cm, white at apex, became darker toward the base, light gray brown at base, cylindrical, slightly expanded at base. Context 0.5–0.7 mm thick at pileus center, milky white, odor none, taste mild. Spore print white.

Basidiospores (6.8) 7.2–8.3 × (4.8) 5.0–5.9 μm, *Q* = 1.37–1.41, (*n* = 30), elliptical, smooth or with verruculose ornamentation, amyloid. Basidia 35–40 × 6.8–8.0 μm, clavate, with four sterigmata. Cheilocystidia and pleurocystidia absent. Trama regular, inamyloid. Pileipellis a cutis consisted of intertwined, thin-walled, dense hyphae, hyphae 6–18 μm wide, cylindrical, not or slightly constricted at the septa, hyaline.

Specimens examined: China: Gansu Province, Zhangye City, Minle Town, 9 August 2018, Jize Xu, HMJU00133; Tianshui City, 8 August 2018 Jize Xu, HMJU00132, HMJU00138.

Habitat and distribution: Scattered on grass. Known from China and Italy.

Notes: *M. microcephala* is included in clade A and belonging to subgenus *Urticocystis*. Differing from interpretations of Fontenla et al. (2013), the stipe of the material from Gansu is slightly longer, and the color of stipe is darker.

***Melanoleuca nivea*** Métrod ex Boekhout, Persoonia 13: 417. 1988 (Figures 7E,F, 9N–Q, 10K,L, 11E–H).

Pileus 3.0–5.0 cm diameter, convex to plane, mostly with low broad umbo, white to pale gray-brown, sometimes with some ochraceous spots, surface smooth, glabrous, slightly viscid when moist, margin somewhat inflexed and slightly exceeding. Lamellae emarginate to adnate, ventricose, thin, up to 8 mm wide, whitish or pale cream, 13–15 lamellae/cm in the edge of the pileus, with intercalated lamellulae, entire to minutely flocculose edge. Stipe 3–5.5 × 0.4–0.8 cm, whitish to pale grayish beige, finally becoming grayish and brownish toward base, cylindrical, somewhat broadening toward base, longitudinally striate, whitish pruinose, glabrous in lower parts when matured. Context 0.4 mm thick at pileus center, whitish, brown to orange-brown, smell faint, somewhat rancid, taste weak, unpleasant. Spore print yellowish white.

Basidiospores 6.5–8.3 (9.1) × 4.1–5.0 μm, *Q* = 1.52–1.98, (*n* = 30), elongate, moderately densely ornamented with rather coarse amyloid warts. Basidia 23–33 × 7–9 pm, clavate, with four sterigmata. Cheilocystidia (35) 40–65 × 9–15 (20) μm, fusiform, partly tending to lageniform, mostly with the apex acute and encrusted by crystals. Pleurocystidia similar to cheilocystidia. Trama regular, inamyloid. Pileipellis an ixocutis composed of radially arranged, thin-walled hyphae, hyphae 5–8 μm wide, cylindrical, hyaline.

Specimens examined: China: Liaoning Province, Huludao City, Bailang Mountain, 28 July 2016, Jize Xu, HMJU00148.

Habitat and distribution: Single on the ground of forest. Known from China and Netherlands.

Notes: *M. nivea* is placed in clade B and belonging to subgenus *Melanoleuca*. Differing from holotype (Boekhout, 1988), the Chinese collections produce a white to pale gray-brown pileus and darker colored stipe.

***Melanoleuca pseudopaedida*** Bon, Docums Mycol. 20: 58. 1990 (Figures 7D, 8A–C, 9A–C, 10M–O).

Pileus 2.0–3.6 cm diameter, plane to depressed at disc, brown to fuliginous, becoming pale, some with white spots, surface smooth, glabrous, slightly viscid when moist, margin slightly incurved, slightly cracked. Lamellae adnate to emarginate, pale gray-brown, 16–18 lamellae/cm in the edge of the pileus, with intercalated lamellulae, edges entire, whitish pruinose. Stipe 2.5–4.5 × 0.3–0.5 cm, pale at apex, brown at center, cylindrical, longitudinally striate, hollow when matured. Context 0.5–0.7 mm thick at pileus center, dirty beige, odor none, taste absent. Spore print white.

Basidiospores (6.7) 7.0–8.4 (8.7) × 5.0–6.0 μm, *Q* = (1.26) 1.30–1.58, (*n* = 30), elliptical to subovoid, densely ornamented with amyloid warts. Basidia 24–30 × 6.8–8.0 μm, clavate, with two or four sterigmata. Cheilocystidia and pleurocystidia absent. Trama regular, inamyloid. Pileipellis tomentum, a cutis composed of thin-walled hyphae, hyphae 6–14 μm wide, cylindrical, not or slightly constricted at the septa, hyaline.

Specimens examined: China: Qinghai Province, Haixi State, Wulan Town, 7 August 2018, Jize Xu, HMJU00155.

Habitat and distribution: Solitary, on grass. Known from China and France.

Notes: *M. pseudopaedida* is included in clade A and belonging to subgenus *Urticocystis*. Differing from holotype (Bon, 1990), the lamellae of the Chinese collections are whitish pruinose and the color is paler.

### Phylogenetic Analysis

**FIGURE 12:**
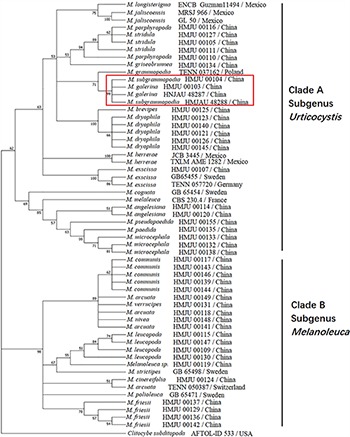
Maximum likelihood tree based on analyses of the ITS and nrLSU sequence data with *Clitocybe subditopoda* as the outgroup.

For the ITS-nrLSU ML analysis, 110 sequences relating to 29 species were added. The ML tree represented as Figure 12 shows detailed results with high bootstrapping values. Phylogenetic analysis produced two Clades: A and B. Clade A was formed in I node, with the bootstrapping value of 81%, while Clade B was formed in II node, having a bootstrapping value of 100%. *Melanoleuca galerina* and *M. subgrammopodia* were independently separated in III node, with a bootstrapping value of 87%. Both *M. galerina* and *M. subgrammopodia* were included in Clade A.

### Divergence Time Estimation

**FIGURE 13:**
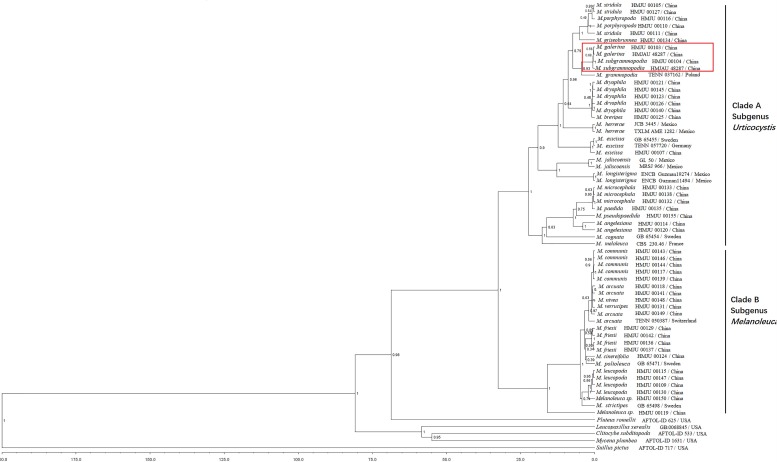
Maximum clade credibility (MCC) tree based on BEAST analyses.

A combination of ITS and nrLSU sequences were used to estimate the divergence time of *Melanoleuca*. The MCC tree represented in Figure 13 shows two diverged clades in *Melanoleuca* since 33.31 million years ago. Clade A contains 18 independent species: *M. griseobrunnea*, *M. pseudopaedida*, *M. microcephala*, *M. galerina*, *M. subgrammopodia*, *M. angelesiana*, *M. porphyropoda* X. D. Yu, *M. stridula* (Fr.) Singer, *M. dryophila* Murrill, *M. brevipes* (Bull.) Pat., *M. exscissa* (Fr.) Singer, *M. paedida* (Fr.) Kühner & Maire, *M. grammopodia* (Bull.) Murrill, *M. herrerae* Sánchez-García & J. Cifuentes, *M. longisterigma* Sánchez-García & J. Cifuentes, *M. jaliscoensis* Sánchez-García, J. Cifuentes & Guzm.-Dáv., *M. cognata* (Fr.) Konrad & Maubl. and *M. melaleuca* (Pers.) Murrill. Clade B contains 9 species: *M. cinereifolia*, *M. communis*, *M. nivea*, *M. leucopoda* X. D. Yu, *M. strictipes* (P. Karst.) Jul. Schäff., *M. polioleuca* (Fr.) Kühner & Maire, *M. friesii* (Bres.) Bon, *M. arcuata* (Bull.) Singer and *M. verrucipes* (Fr.) Singer. Furthermore, *M. galerina* and *M. subgrammopodia* diverged 11.48 million years ago.

## Discussion

In this study, two new species from northeastern China were described in detail. Based on Boekhout (1988) *M. galerina* belongs to the subgenus *Melanoleuca* and *M. subgrammopodia* belongs to the section *Grammpodiae* in the subgenus *Urticocystis*. However, using phylogenetic analyses, the species in our investigations are divided into two Clades. The species in clade A are with urticiform cystidia or without cystidia, and in clade B have macrocystidia. This result was corroborated by Vizzini et al. (2011) whereby the authors divided the genus *Melanoleuca* into two subgenera: the subgenus *Urticocystis* mainly included the species with urticiform cystidia or without cystidia, while the subgenus *Melanoleuca* mainly included the species with macrocystidiate. Therefore, in this study, our phylogenetic analyses of *Melanoleuca* species using combined ITS and nrLSU sequences suggests that *M. galerina* and *M. subgrammopodia* are new species belonging to the subgenus *Urticocystis*.

In our investigations, the results of divergence time were in line with phylogenetic analysis, and supports that *M. galerina* and *M. subgrammopodia* are new species. In addition, results also indicate that divergence time of a lineage could be used as a criterion for ranking taxa (Zhao et al., 2016; Chen et al., 2017). But, the selection of proper calibration points provided by fossils is an important aspect. Moreover, reliable calibration points can provide evidence for divergence time (Feng, 2012).

## Data Availability Statement

The datasets generated can be found in NCBI, accession numbers can be found in Table 1.

## Author Contributions

JX wrote the manuscript. JX, XY, ML, and CZ carried out the experiments. JX, CZ, and JH collected the specimens. LG and YL designed the experiments. OM had a contribution in revising the manuscript and taking SEM photographs.

## Conflict of Interest

The authors declare that the research was conducted in the absence of any commercial or financial relationships that could be construed as a potential conflict of interest.

## Footnotes

1 http://www.indexfungorum.org (accessed June 10, 2019).

2 http://www.phylogeny.fr/one_task.cgi?task_type=gblocks

3 https://mega.co.nz/

4 http://beast2.org/

5 https://github.com/beast-dev/tracer/releases

6 http://tree.bio.ed.ac.uk/software/figtree/
